# Parents’ actions, challenges, and needs while enabling participation of children with a physical disability: a scoping review

**DOI:** 10.1186/1471-2431-12-177

**Published:** 2012-11-08

**Authors:** Barbara Piškur, Anna JHM Beurskens, Marian J Jongmans, Marjolijn Ketelaar, Meghan Norton, Christina A Frings, Helena Hemmingsson, Rob JEM Smeets

**Affiliations:** 1 Faculty and Health Care, Centre of Research Autonomy and Participation for persons with a chronic illness, Zuyd University, Nieuw Eyckholt 300, Heerlen, DJ 6419, the Netherlands; 2 Department of Occupational Therapy, Faculty of Health and Care, Zuyd University, Nieuw Eyckholt 300, Heerlen, DJ6419, the Netherlands; 3 Department of Rehabilitation Medicine, CAPHRI, School for Public Health and Primary Care, Faculty of Health, Medicine and Life Sciences, Maastricht University, P.O. Box 616, Maastricht, MD6200, the Netherlands; 4 Department of General Practice, Faculty of Health, Medicine and Life Sciences, Maastricht University, P.O. Box 616, Maastricht, MD6200, the Netherlands; 5 Rudolf Magnus Institute of Neuroscience and Center of Excellence for Rehabilitation Medicine, University Medical Center Utrecht and Rehabilitation Center De Hoogstraat, Rembrandtkade 10, Utrecht, TM3583, the Netherlands; 6 Department of Special Education, Faculty of Social Sciences, Utrecht University, PO Box 80140, Utrecht, TC3508, the Netherlands; 7 Department of Neonatology, Wilhemina Children's Hospital, University Medical Center Utrecht, Utrecht, AB 3508, the Netherlands; 8 Partner of NetChild, University Network for Childhood Disability Research in the Netherlands, Utrecht, the Netherlands; 9 Department of Social and Welfare Studies (ISV), Faculty of Health Sciences, Linköping University, Norrköping, 601 74, Sweden; 10 Aachener Laienhelfer initiative e.V, Sophienstrasse 15, Aachen, 52070, Germany; 11 Adelante centre of expertise in rehabilitation, Zandbergsweg 111, Hoensbroek, CC6432, the Netherlands

**Keywords:** Participation, Social participation, Physical disability, Children, Parents, Scoping review

## Abstract

**Background:**

Pediatric rehabilitation considers Family-centered service (FCS) as a way to increase participation of children with a physical disability in daily life. An important principal is that parents greatly contribute to their child’s participation at school, at home, and in the community. However, it is unclear what kind of information is available from literature about what parents actually do to support their child’s participation and what problems and needs they experience? Hence, the aim of this study was to provide an overview of the actions, challenges, and needs of parents in enabling participation of their child with a physical disability that is neurological and non-progressive in nature.

**Methods:**

Scoping review with extensive literature search (September 2011) and a thematic analysis to synthesize findings.

**Results:**

Fourteen relevant articles revealed two major themes: ‘parents enable and support performance of meaningful activities’ and ‘parents enable, change and use the environment’. Each theme holds a number of actions (e.g. choosing the right type of meaningful activities for facilitating social contacts) and challenges (e.g. negative attitudes of other people). Less information is available about the needs of parents.

**Conclusions:**

This study indicates that parents apply a broad range of strategies to support participation of their children. They experience many challenges, especially as a result of constraints in the social and physical environments. However, this review also shows that little is known about needs of parents in facilitating participation. As Family-centered service (FCS) philosophy is all about the needs of the child and the family, it is essential to further investigate the needs of the parents and to understand if and to what extent they wish to be supported in enabling their child’s participation in daily life.

## Background

The concept of participation is important in the field of childhood disability [1]. Participation has been defined by the International Classification of Functioning, Disability and Health (ICF) as “a person’s involvement in life situations” [2]. For children, involvement includes participation in everyday activities, such as recreational, leisure, school, and household activities [3]. Participation is an important outcome for the health of adults and children [4-7]. Furthermore, children’s participation at home, at school, and in the community relates to well-being, quality of life, and development [5,8-10]. Several authors use the term social participation for participation, emphasizing the importance of engagement in social situations [11-13]. Through participation in different social contexts, children gather knowledge and skills needed to interact, play, work, and live with other people [14,15].

Several publications [16-18] consider participation to be a fundamental right for children; the more meaningful a child’s participation is, the more he or she develops a sense of identity and becomes confident and competent to deal with peers, adults, and the extended society. For these reasons, the enhancement of participation is a key topic of the revised European Social Charter [19]. The ICF operationalizes participation as what an individual does in his or her current environment and the individual’s ability to execute a task or an action in real life situations [2]. Others [20,21] stressed that in addition to performance, engagement in activities is part of participation. For example, several studies [22,23] show people might consider themselves to be engaged and participate in activities without actually performing them.

The degree of participation of children with a physical disability is associated with several variables, such as gross motor function, communicative skills, and environments [24]. Children with a physical disability experience participation restrictions. They participate less frequently in almost all activities compared to children without physical disabilities [25,26]. As a result, they have decreased opportunities building relationships and often feel socially isolated [27-29]. It is commonly known that accessible or accommodating facilities enable participation of children with physical disabilities [30].

The support of the social environment is equally important: parents, peers, teachers, community-members, and friends. Parents, in particular, greatly influence participation at school, at home and in the community [31]. They undertake many actions to improve their children’s participation in daily life [31,32]. Understanding the actions of parents and also their challenges and needs will contribute to how society can support these parents and thereby enable the participation of children with physical disabilities. Pediatric rehabilitation, aiming for optimal participation [33,34], could benefit from this understanding to improve Family-centered services (FCS). In FCS, the family is seen as an expert on the child’s abilities and needs, and professionals work in partnership with the family [34,35]. Pediatric rehabilitation considers FCS as a way to increase participation of children with a physical disability in daily life.

However, it is unclear what kind of information is available in literature about what parents live through, do, and what kind of problems and needs they have in supporting their child’s participation? For these reasons, a scoping review was conducted in order to systematically map the research done in this area, as well as to identify any existing gaps in knowledge. Scoping review can be undertaken as a stand-alone project, especially where an area is complex or has not yet been comprehensively reviewed [36]. The following research question was formulated: What is known from the literature about parents’ actions, challenges, and needs while enabling participation of their children with a physical disability? It was decided to focus on parents of children belonging to the major group of pediatric rehabilitation clients in the Netherlands, as well as, in Europe [37]; children with physical disabilities that are neurological and non-progressive in nature (e.g. Cerebral palsy, Spina bifida).

## Methods

### Scoping reviews

For this review, the methodological framework of Arksey & O’Malley [38] was applied. This framework consisted of the following main phases: design and search for relevant studies, selection of studies, charting the data, and the collation, summarization, and reporting of the results. Similar steps used for mixed-method systematic reviews were followed [39]; typically in scoping reviews, the appraisal and inclusion of evidence is not limited by the methodological quality of that evidence [38,40].

### Search terms and search strategies

This review focused on the actions, challenges, and needs of parents having a child between 0-18 years of age with a physical disability resulting from a neurological cause (e.g. cerebral palsy, spina bifida). An initial orientation search was conducted to extract the key search terms. The search strategies used the following formula: parents AND children AND diagnose OR physical disability AND need OR wish OR problem OR action OR strategy AND social participation. Search terms for “parents” included MeSH terms like “parents”, “caregivers”, or “single parent” in combination with free-text terms such as “parenting” or “grown-up”. Search terms for “children” included the MeSH term “child” combined with free-text terms such as “children” or “scholar”. Search terms for “physical disability” included MeSH terms such as “disabled children” combined with free-text terms such as “physical impairment” or “physical dysfunction”. Diagnostic labels were also added to the search process. MeSH terms like “cerebral palsy” or “spinal dysraphism” were combined with free-text terms such as “infantile cerebral paralysis” or “spina bifida”. Expressions that focus on parents’ actions, challenges, and needs were searched with free-text terms like “action”, “challenge”, “demand”, “wish”, “desire”, “need”, or “problem”. MeSH terms like “social participation” and “social environment” were combined with free-text terms like “participation”, “social competence”, or “formal participation”.

During September 2011, the databases for PubMed, Psychology and Behavioral Sciences Collection, and PsycINFO were searched with no restrictions to the publication date. In addition, a manual search of articles in four journals (American Journal of Occupational Therapy, British Journal of Occupational Therapy, Canadian Journal of Occupational Therapy, Scandinavian Journal of Occupational Therapy) together with an Internet browser search (scholar.google.com) using the key search terms (“parents”, “children”, “cerebral palsy [and other diagnoses]”, “physical disability”, “need”, “wish”, “problem”, “action”, “strategy”, “social participation”, “participation”) was conducted to locate and extract any additional publications or grey literature.

### Study selection criteria

A four-stage process was used to identify selection criteria for study reviews. First, because parents’ actions, challenges, and needs were the subject of this scoping review, parents were the primary target population of in the study in order to meet the selection criteria. Further, any studies showing parents’ opinions or experiences, or both, towards the participation of their child with a physical disability were of particular interest in the review process. Second, to limit the scope of the review, studies also had to include only parents of children between 0 and 18 years of age having a physical disability that was deemed non-progressive and of neurological origin (e.g. cerebral palsy, spina bifida). If a study also included parents of children with other kinds of disabilities, that group had to be the minority of the study population. Third, studies were required to focus on those particular actions, challenges, or needs of parents that enabled participation of their children in daily activities at home, at school and the community. Participation could refer to the actual performance of activities or the engagement in activities. A “need” is described as a motivating force that compels action for its satisfaction [41] or a lack of something wanted [42]. An “action” was considered as the process of doing something, especially when dealing with a problem or difficulty [43]. A “challenge” is often threatening, provocative, stimulating, or inciting [44] and can be perceived as a problem that is defined as a gap between the existing state and a desired state [41]. Fourth, no restrictions were imposed regarding the type of design or year of publication for studies reviewed. The original language of each study, however, was limited to English, German, and Dutch.

### Study selection

Three reviewers (BP, MN, CAF) independently evaluated and scored each study using the inclusion criteria described above. They recorded their evaluation by labeling each as either relevant (R), irrelevant (I), doubtful (D), or double (DO). Next, study abstracts were divided into three equal groups and assessed independently by the three reviewers. This step was followed by a cross-check by BP of 40 abstracts to check for consistency. Next, full-text articles were reviewed by BP and cross-checked by MN and ACF. In case of disagreement, a fourth person (AJHMB) stepped in to reach a consensus.

### Charting the data

In scoping reviews, the process employed in the selection and charting of data generally includes studies that use mixed methodologies. This requires a subsequent synthesis, grounded within interpretative, narrative, and descriptive analytical methods [40,45-47]. A data-charting form was developed to determine which variables to extract. This form provided for descriptive entries (e.g., study design) and for specific narrative information (e.g., actions, challenges, needs in relation to supporting participation). The three reviewers independently charted the data and discussed the results.

### Collating, summarizing, and reporting the results

Data collation and summarization was done in two steps, as recommended methodological procedures found in the literature [38,40]. A descriptive summary of each study was made, consisting of the following elements: author, year, country, aim of the study, study design and population, and principal findings (see Table 1). Narrative synthesis was used to summarize evidence from 3 streams (quantitative descriptive, mixed methods, and qualitative studies) involving a qualitative, thematic analysis [46-49]. Codes and labels were formatted in the findings section of each article. Labels were ordered and discussed by the three reviewers. This resulted in themes that define the scope of the study, including the reviewers’ interpretation of the data.

**Table 1:** Descriptive summary of the relevant studies

	**Author, Year, and Country**	**Aim of the Study**	**Study Design**	**Description of Study Population**	**Focus on Social Participation, Participation, or Activity**
50*	Heah T, Case T, McGuire B, Law M	Parent/child experiences regarding	Qualitative research	8 parents (1 father, 7 mothers) and 8 children (5 - 16 years; 5 boys, 3 girls) with physical disability (neurological and/or musculoskeletal disabilities)	Participation in everyday occupations
	2007	- what successful participation means to children and families	Phenomenological approach		
	Canada	- what support and what hinders participation	Semi-structured interviews		
51	Antle BJ, Mills W, Steele C,Kalnins I, Rossen B	Gain insight into parental health promotion efforts within the family context where there is an adolescent with a physical disability	Qualitative research	15 parents (11 two-parent and 4 single parent families) and 15 children (11-16 years; 13 boys, 2 girls) with a diagnosis of physical disability (Cerebral palsy: 7, Spina Bifida: 3, Muscular dystrophy 3, other conditions:2)	Play, leisure, and educational activities
	2007		Long interview Method		
	Canada				
52	Missiuna C, Moll S, King S, King G, Law M	To explore parent perspectives regarding the early experiences of their children with Developmental Coordination Disorder	Qualitative research	13 parents of children with Developmental Coordination Disorder (6-14 years; 10 boys, 3 girls)	Play, leisure, and educational activities
	2007		Phenomenological approach		
	Canada		In-depth interviews		
53	Missiuna C, Moll S, Law M, King S, King G	Explore the early experiences and participation patterns of children with Developmental Coordination Disorder, as perceived and reported by the parents.	Qualitative research	13 parents (2 fathers, 13 mothers; 13 two-parent families) and 13 children (6- 14 years; 10 boys, 3 girls) with diagnosis of Developmental Coordination Disorder	Play, leisure, and educational activities
	2006		Phenomenological approach		
	Canada		Semi-structured interviews		
54	Bedell GM, Cohn ES, Dumas HM	Describe parents’ perspectives about the strategies they use to promote social participation of their school-age child with Acute Brain Injury	Qualitative research	16 Parents (3 fathers and 16 mothers; 15 two-parent and 1 single parent families) and children (5-15 years; 6 boys, 10 girls) with Acute Brain Injury	Social participation
	2005		Semi-structured interviews (content and constant-comparison analysis)		
	USA				
55	Huang Y P, Kellett U, St. John W	Describe a range of challenging care-giving experiences of Taiwanese mothers providing for their children with cerebral palsy (Cerebral palsy).	Qualitative research	15 Mothers of children with Cerebral palsy (8 months- 14 years)	Activities of daily living and educational activities
	2011		Hermeneutic Phenomenological		
	Taiwan				
56	Bennett K, Hay D	Test the hypothesized model to determine individual, family, and teacher characteristics associated with social skills development in children with physical disabilities	Quantitative research	212 parents and children (5-12 years) with a physical disability; 170 teachers in mainstream schools	Educational activities
	2007		Descriptive study		
	Australia				
57	Hewitt-Taylor J	Reports the parents views of their children’s experiences in relation to these activities	Qualitative research	14 parents and 14 children (18 months- 18 years)	Play and educational activities
	2008		Semi-structured interviews		
	UK				
58	Buran CF, Sawin K, Grayson P, Criss S	Survey the parents of children with Cerebral palsy and report their needs for information, services, and access to treatment	Quantitative research	475 families receiving services at a multidisciplinary Cerebral palsy Clinic; children (mean age 8 years 11 months; 266 girls, 209 boys)	Recreational activities
	2009		Descriptive study		
	USA				
59	Meehan DR	Describe the experience of mothering a 3-6 year old child with hemiparesis	Qualitative research	5 Mothers (5 two-parent families) and children (3-6 years; 4 boys, 1 girl)with a diagnosis of hemiparesis	Leisure activities
	2005		Phenomenological approach		
	USA		Interviews		
60	Lawlor K, Mihaylov B, Welsh S, Jarvis S, Colver A	Identify features of environments that facilitate or restrict participation	Qualitative research	12 Parents (3 fathers, 5 mother, 1 grandmother) and children (5-17 years; 6 boys and 6 girls) with Cerebral palsy	Participation as defined by the International Classification of Function
	2006		In-depth interviews		
	UK				
61	Vogts N, Mackey A, Ameratunga S, Stott NS	To pilot the use to the Craig Hospital Inventory of Environmental Factors (CHIEF) questionnaire to ascertain information regarding barriers to participation	Mix-methods: Quantitative data with Qualitative feedback	32 Parents and children (6-16 years, 15 boys and 7 girls) with Cerebral palsy	Participation as defined by the International Classification of Function
	2010				
	New Zealand				
62	Hewitt-Taylor J	Gain understanding of parent’s views regarding the social inclusion of their children who have complex and continuing health needs	Qualitative research	14 parents(2 fathers, 12 mothers; 12 two- parent families, 2 single parent families) and 14 children (18 months- 18 years) with complex health needs (learning problems as well as health problems)	Leisure activities
	2009		Semi-structured interviews		
	UK				
63	Palisano RJ, Almarsi N, Chiarello LA, Orlin MN, Bagley A, Maggs J	Identify (1) differences in the number and types of family needs based on the child’s age and gross motor function level; (2) the most frequent expressed family needs; and (3) needs that differ based on gross motor function level	Quantitative research Cross-sectional analytical design	501 parents (389 mothers, 59 fathers, 25 grandmothers, 28 others) and children (2-21 years)with Cerebral palsy	Physical activities
	2009				
	USA				

* The numbers in Table 1 correspond with the numbers in the reference list and the numbers in Figure 2.

## Results

**Figure 1:**
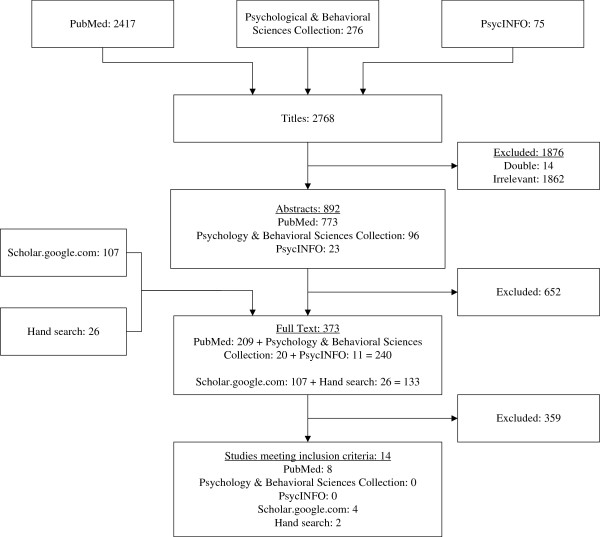
Flowchart of study selection.

In total, 2,768 articles were identified as potentially relevant from the search in the following databases: PubMed, Psychology and Behavioral Sciences Collection, and PsycINFO. After screening the titles, 892 articles appeared to be relevant. Screening of abstracts resulted in 240 potentially relevant articles. In addition to these, the grey literature and manual search yielded an additional 133 articles. All 373 articles were then evaluated on a full-text level, resulting in a final total of 14 articles relevant for charting. (See Figure 1).

### Descriptive summary of the studies

The majority of the 14 relevant articles remaining after application of selection criteria were conducted in Canada and the United States followed by the United Kingdom (See Table 1). Parents of children aged between 0 – 18 years had participated in the included studies. All articles had been published between 2005 and 2011. Ten articles consisted of qualitative studies; three of quantitative descriptive studies, and one article used a mixed method approach. The study population in eleven articles included parents having a child afflicted with a physical disability and children themselves. In two articles, only the parents of a child with a physical disability were included, and in one article, the study population involved the parents, the child with a physical disability, and their teacher.

### Narrative summary of the studies

**Figure 2:**
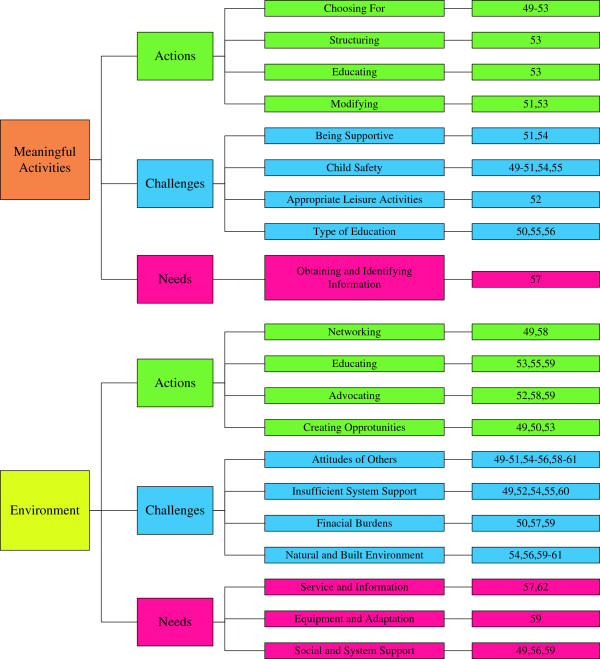
Flowchart of results based on thematic analysis.

Results of the qualitative thematic analysis were organized along two major themes: (1) parents enable and support performance of meaningful activities, and (2) parents enable, change, and use the environment. In Figure 2, the actions, challenges, and needs are graphically presented.

#### Parents enable and support performance of meaningful activities

This theme is about actions, challenges, and needs of parents in relation to helping their child with a physical disability to engage or be involved in meaningful activities in order to enable participation. Here, the term meaningful relates to the subjective perception of parents about the meaning of activities regarding participation of the child. Five out of 14 studies demonstrated the following actions or strategies: “choosing for”, “structuring”, “educating”, and “modifying” activities.

“Choosing for” refers to the action by which parents make choices for or with the child about the kinds of activities in which he or she will engage. Heah et al. [50] found that parents had strong convictions that their children should experience a variety of activities in order to choose those that are particularly meaningful. Parents attributed different meanings to activities: having fun, feeling successful, doing and being with others, and doing things yourself [50]. In the study of Antle et al. [51], parents stressed the importance of exploring physical activities in order for their child to stay healthy and to develop self-confidence and discipline. Missiuna et al. [52] provided similar examples of parents who decided to enroll their child with a physical disability in recreational activities or in team sports in order to be better engaged with their peers. Occasionally, however, parents chose to limit or avoid sports activities if these activities proved to be too demanding in relation to their child’s physical abilities, or to reduce the frustration levels of both parents and their child [51,53,54]. In some cases, mainstream education includes activities that are too demanding for a child with a physical disability. In one example [50], when parents noticed their child was at risk of falling behind his or her peers, they choose another type of educational institution.

“Structuring” refers to the way in which parents apply strategies to organize the day so that enough time is left for a child to engage in meaningful activities. One article addressed this specific action. In a study of Bedell et al. [54], in order to promote participation, mothers composed strategies that incorporated the daily needs of the family with that of the child by orchestrating activities and routines that enhanced the child’s participation and experience. Specific strategies were not further described.

“Educating” is about teaching and coaching a child on how to solve problems while performing new or difficult activities. One study [54] showed examples of how parents enabled their child’s participation by using several types of cognitive and behavioral strategies to improve performance. Modeling, showing, or describing the process, using trial and error, or repeating activities in the same or different contexts, were among the strategies found to be useful and valuable. Parents educated their child about how to deal with peers at school who engaged in behaviors, such as teasing [54]. Further, parents stimulated the learning process by setting limits, by being very consistent, or by using cues that supported their child’s ability to perform meaningful activities.

“Modifying” stands for adaptations of activities to support the child’s independence and social interaction. Missiuna et al. [52] provided examples by which parents helped their child perform everyday activities more effectively. One such example – putting on a jacket for playing outdoors – was facilitated by buying clothes without buttons. Parents indicated that this was especially important when performance was interfering with a child’s routine situations during school time. In another example given by Bedell et al. [54], parents broke down difficult household activities into smaller tasks, such as involving their child in parts of the laundry process.

While creating opportunities for their child to engage in meaningful activities, parents experienced various challenges. These included “being supportive in a correct manner”, “coping with child safety”, “choosing the most appropriate leisure activities”, and “selecting the best type of education”. Such types of challenges were discussed in six of the 14 studies.

Two studies [52,55] illustrated the challenge of “being supportive in a correct manner” during performance of difficult activities. Parents did not know how to help their child once he or she became angry while doing a homework assignment [52]. Huang et al. [55] showed that parents struggled between encouraging their child’s independence versus maintaining their responsibilities as parents.

“Coping with child safety” is another important challenge for parents. Studies by Heah et al. [50] and Missiuna et al. [52] demonstrated parents’ vigilance when children went out with friends, such as playing in the park or attending a party. The more severe the child’s disability, the more alert and involved his or her parents were [56]. Often, parents became overprotective, as described in the studies of Antle at al. [51] and Huang et al. [55].

Parents faced other challenges in addition to home and school activities, such as “choosing the most appropriate leisure activities” that fit the child’s abilities while bringing a sense of accomplishment. For example, Missiuna et al. [53] described parents’ struggles with physical leisure activities. Parents sought to avoid tasks in which the child experienced repeated failure, even taking the risk that by withholding their child from team sports, opportunities for peer connections might become more limited.

“Selecting the best type of education” to support their child’s future is yet another challenge confronting parents. The study reviews indicated that parents believed that an appropriate education was an important condition for future success. Antle et al. [51] found many worries among parents about the future of their children: worries about having a career, about being financially independent, and about being able to live on their own and having friends. While some parents believed that a mainstream school is the best way to succeed in society, others were afraid their child would be too different from their peers in such a school system [56,57].

Only one study [58] addressed the needs of parents in “identifying and obtaining information” about meaningful activities for their child. Concerns encompassed the need of obtaining more information about the availability of recreational and entertainment activities, as well as information about education and special education.

#### Parents enable, change and use the environment

This theme is about actions, challenges, and needs of parents while using, enabling, and changing the social and physical environment at home, school, and in the community to support the participation of their child with a physical disability. In addressing this theme, seven studies described actions or strategies: “networking”, “educating”, “advocating”, and “creating opportunities”.

“Networking” refers to the establishing of connections with people with similar experiences, who understand the parents’ situation, and who are willing to support them. Heah et al. [50] and Meehan [59] illustrated that, in connecting with other parents of children with a disability, parents became more informed about community programs and suitable activities for their children. In addition, these connections provided parents with a feeling of belonging to a group with shared interests [59]. Further, Heah et al. [50] reported that parents identified and organized a wide range of social support (friends, family, or support workers) with the aim of increasing the participation of their child in community activities and social interactions. For example, one set of parents engaged a support worker to escort their child outside of the house to be with friends.

“Educating” is defined as the giving of instructions to others on how to support the activity performance of their child. Explaining to a teacher how to make educational activities more suitable to their children [56], or providing a teacher with written strategies are two examples of how parents helped to educate school staff [54]. Similar strategies were used for extended family members and services, such as respite care [60].

“Advocating” refers to the competing of resources, supports, and services within the system. Examples are given by Missiuna et al. [53], Meehan [59], and Lawlor et al. [60], in which parents actively advocated for additional services at school, like the presence of a teacher-assistant while taking an exam, or they spoke up for their child’s best interest, or they fought for extra resources during leisure activities. To get appropriate support for their child, parents promote awareness about the child’s abilities, strengths, and needs in an attempt to change peoples’ attitudes toward their disability.

“Creating opportunities”, as an action, means the creation of events by parents in order to shape opportunities for their child to get acquainted with other children. Heah et al. [50] and Antle et al. [51] described how parents often organized meetings with others to create opportunities and situations for their child to meet friends. Parents worried about their child being alone at parties. To cope, some parents held dual parties: one for themselves and one for the children [51]. Additionally, Bedell et al. [54] showed that parents purposefully selected certain peers to visit or play with their child after school in order to increase the chances for developing a solid friendship.

Twelve studies addressed several parents’ challenges related to the theme of “enable, change, and use of the environments”. These comprised challenges such as the “attitudes of others”, “insufficient system support”, “financial burdens”, “lack of time”, and “barriers in both the natural and built environments”.

The “attitudes of others” refers to the experience by which parents faced negative attitudes of other children or adults towards their child with a physical disability. The fact that parents have to deal with these attitudes is shown by the many worries and concerns that were expressed in several of the 14 research studies. Parents worried that their child would not be accepted by peers, or would be teased or hurt emotionally or physically [50,51,56,57,59]. Missiuna et al. [52] and Vogts et al. [61] also found that parents harbored concerns about their child being criticized by their teacher for not performing at the expected level. Negative attitudes, comments, and prejudice of others influenced the joy of being together as a family and thereby, impacted the participation of their child [55,60,62].

“Insufficient system support” pertains to the challenges stemming from unsupportive social structures. Six research papers addressed challenges related to the school system. Bennett & Hay [56] documented parents’ concerns about the lack of help their children received from teachers and about the insufficient qualifications those teachers had in educating children with physical disabilities. Furthermore, studies by Vogts et al. [61], Missiuna et al. [53], Heah et al. [50], and Huang et al. [55] indicated that support and help at school was clearly not sufficient for children with disabilities. Additionally, in the study of Heah et al. [50], parents expressed that community programs do not provide enough opportunities for children with disabilities to play with others.

“Financial burden” illustrates the challenges faced by families in dealing with monetary constraints to support the participation of their child. Antle et al. [51] reported that parents with low incomes experienced stress when they lacked the resources necessary to enroll their child in recreational activities. Conversely, other parents discovered that their income was too high to receive financial support from the government and too low for addressing their child’s needs [58]. Parents were often unaware of other financial support programs to which they were entitled [60].

A “barrier in the natural and built environment” refers to the physical accessibility of buildings and public places. Hewitt–Taylor [62] gave examples of challenges confronting parents in non-user-friendly shops, cinemas, and public toilets. Similar challenges were also experienced in parks, public transport, and parking facilities -- all noted as not being user-friendly for children with a physical disabilities [57,60-62]. Also, schools, playgrounds, and leisure facilities in neighborhoods were often inaccessible to children with a physical disability [55,61]. These environmental barriers present many challenges for parents to find appropriate outdoor activities for their child to play with other children.

Five studies addressed parents’ needs regarding enabling environments: “service and information”, “equipment and adaptations”, and “social and system support”.

“Service and information needs” refers to parents’ needs for available centers and services in the community suitable for providing leisure activities for children with a physical disability. Palisano et al. [63] showed that parents sought out extra support persons or services to help them locate appropriate community camps, sports, recreational, social, and leisure activities. Furthermore, Buran et al. and Palisano et al. [58,63] illustrated how parents require more written information than is generally available about services available in their community.

“Equipment and adaptation” refers to the need for adequate equipment that is designed to support independence and participation in activities, while reducing the level of care. A study by Lawlor et al. [60] referred to the parents’ needs for more user–friendly designs of transport systems and parking facilities, as those facilities are vital for attending leisure activities, school sessions, and hospital appointments.

“Social and system support” refers to the needs of parents for more expansive social networks and accessible leisure centers to enable the participation of their children. In several studies [50,57,60] parents also expressed the need for extra support from grandparents by bringing their child to leisure activities or to school, ensuring that parents would be able to continue to work.

## Discussion

The purpose of this scoping review was to explore what is known in the literature about parents’ actions, challenges, and needs while enabling participation of their children with a physical disability. Fourteen articles, all published after 2004, were included in the review.

The findings of this scoping review reveal that parents of children with a physical disability use, enable, and change the social and physical environment to facilitate participation. Further, they facilitate their child to engage or perform in meaningful activities. The most cited *actions* in the 14 studies reviewed are “choosing for” meaningful activities for their child, “advocating” for the child, “educating” the social environment, and “networking” with other people. The present study illustrates further that, in supporting participation, parents often face *challenges* in the environment such as “attitudes of other people”, “insufficient system support”, and “barriers in both the natural and built environments”. Parents of children with a physical disability frequently experience difficulty in finding suitable educational systems and meaningful activities for their children that support their child’s participation outside of the home. Ten out of 14 articles demonstrated parents’ challenges with the “attitudes of other people” at school and in the community. Only a small number of studies discussed parents’ *needs* in enabling participation; “social and system support” needs are the most often reported, followed by the needs for “services and information”.

There is a debate in the literature about the concept of participation [11,64]. The International Classification of Functioning, Disability and Health (ICF) definition of participation used in this scoping review has been criticized in the literature [20,65,66] for not including a personal meaning. Perenboom & Chorus [67] stress the importance of fulfillment of personal goals and societal roles for participation, although their study found that several measurement instruments for participation only focused on the actual performance of activities. In the present study, it is unclear whether the meaning parents attribute to certain activities is congruent with the personal meaning their child is experiencing. Furthermore, Perenboom & Chorus [67] argue that being autonomous to some extent or being able to control your own life, is part of participation. Participation can exist even if one is not actually doing things themselves. Some authors [68,69] assigned the importance of engagement and motivation to the definition of participation. In our review, studies were included if they focused on parents enabling participation of their child in daily activities, regardless of whether the child actually performed or was engaged in the activity.

The results of this study may be hampered by limitations. Although a number of free-text and MeSH terms were used for addressing physical disabilities, there is a possibility that non-reviewed studies may have used other terms with similar intent or meaning. In addition, we may have missed studies due to database selection bias. This review did not specifically focus on literature in the fields of education or special education. Nevertheless, there is only a small chance that some studies were missed, as we conducted extensive manual searching (including the literature lists of included articles) and additional searches using scholar.google.com. This study does not only present a descriptive summary of the findings, but the thematic analysis also led to a synthesis that leads to further understanding of parents’ actions, challenges, and needs. Most studies were conducted in western societies, like the United States and Canada. As social and cultural contexts differ in countries and regions, the results of this study may be influenced by cultural bias.

Similar findings of *actions* have been found in studies with other populations, e.g. parents of children with Down syndrome, young people with epilepsy, and young adults with physical disabilities [70-72]. Bedell et al. [72] gave examples of parents educating others and Reid et al. [70] showed examples of parents advocating for equal rights. In a study of parents’ perceptions of their children with developmental coordination disorder (DCD), Segal et al, [73] found that physical activity is an important facet of social life, as impaired performance could lead to participation restriction. In many of the studies in the present review, parents were greatly concerned with enrolling their child in physical or recreational activities in order to enhance participation.

*Challenges* of parents have also been presented in studies with other populations. Bedell et al. [72] and Reid et al. [70] presented similar challenges of parents integrating their young adults in appropriate leisure activities, changing the attitudes of others, and dealing with insufficient system support. These findings also relate to theories about social stigma, as described by Goffman [74]. Social stigma is a severe social disapproval of, or personal discontent with, a person on the grounds of his unique characteristics distinguishing him from others in society [74]. Negative attitudes have an undesirable effect on children, leading to negative consequences such as low self-esteem and reduced participation [75].

In pediatric rehabilitation, Family-centered service (FCS) is seen as a best practice [34]. FCS requires active family involvement in all stages of the rehabilitation process. Knowledge about what parents do, experience, and need in enhancing their child’s participation is crucial in developing tools and strategies for FCS. The number of studies conducted in this area is still rather low. The 14 included articles indicate that parents do perform many actions and experience many challenges in enhancing participation. Most of these actions and challenges seem to focus on the environment. Altering the environment might be the main determinant of change in enabling childrens’ participation. Professionals engaged in FCS could question whether they pay enough attention on supporting the parents in this endeavor.

This review shows that little information is available about parents needs in supporting participation of their child with a physical disability. As FCS is all about the needs of families, more research is necessary to gain further understanding of what parents really need and how they would like to be empowered in enhancing participation of their child. Furthermore, FCS can be improved if more knowledge is available about the relationship between characteristics of contexts, families, parents and children, and the actions, challenges, and needs of parents.

## Conclusions

This review shows that parents apply a broad range of strategies to support participation of their children. They experience many challenges, especially as a result of constraints in the social and physical environments. However, this review also displays that little is known about needs of parents in facilitating participation. Further investigation into the needs of parents is warranted to understand if and to what extent they wish to be supported in enabling their child’s participation in daily life.

## Abbreviations

IFC: International Classification of Functioning, Disability and Health; FCS: Family-centered services.

## Competing interest

The authors report no conflict of interest. The authors alone are responsible for writing this article, and for its contents.

## Authors’ contributions

BP conceived the study idea and led the design. The review process was led by BP and completed by BP, MN, CAF, and AJHMB. All authors provided insight and contributed considerably to the overall study completion. BP prepared the first draft of the manuscript; the other authors commented on the drafts and provided feedback in the editing process. All authors have read and approved the final manuscript for submission.

## Authors’ information

BP has a master of science and is a specialist occupational therapist in the field of pediatric rehabilitation. She is a researcher of the Center of Research Autonomy and Participation for persons with chronic illness and a senior lecturer of the Department of Occupational Therapy at Zuyd University of Applied Sciences. Currently, she working on her PhD at Maastricht University in the Netherlands and is an active member of the University Network for Childhood Disability Research (NetChild). AJHMB is a physiotherapist, registered epidemiologist, and an associate professor of the Center of Research Autonomy and Participation for persons with chronic illness at Zuyd University of Applied Sciences. She is the coordinator of a large number of research projects regarding client-centered care, care innovations and implementation, and clinimetrics. MJJ is a professor of Special Education at Utrecht University. She has previously supervised projects investigating participation of young children with a physical disability; she is a member of NetChild, working in close collaboration with CanChild, Centre for Childhood Disability Research in Hamilton, Canada. MK is an associate professor of Neurology and Neurosurgery, Rehabilitation and Sport Medicine, a programme leader (Pediatric Rehabilitation), and a programme coordinator (National Research Programme on Pediatric Rehabilitation, Netherlands). MN has a master of science in occupational therapy and is a researcher of the Center of Research Autonomy and Participation for persons with chronic illness at Zuyd University of Applied Sciences. CAF is an occupational therapist in Germany and a research assistant of the Center of Research Autonomy and Participation for persons with chronic illness at Zuyd University of Applied Sciences. HH is a professor of occupational therapy at Linkoping University focusing on participation and disability in everyday life and a project leader of studies that focus on the facilitators and barriers that young people encounter in their environment. RJEMS is professor of Rehabilitation at Maastricht University and the Chairman of the Department of Rehabilitation Medicine at Maastricht University Hospital. He is also the manager of the Adelante Expertise Centre of Rehabilitation Medicine and Audiology.

## Pre-publication history

The pre-publication history for this paper can be accessed here:

http://www.biomedcentral.com/1471-2431/12/177/prepub
